# Synergistic effects of surface-enhanced Raman spectroscopy and enzyme-linked immunoassays in diagnosis of Alzheimer's disease, mild cognitive impairment, and late-life depression

**DOI:** 10.3389/fneur.2025.1615457

**Published:** 2025-07-02

**Authors:** Xi Mei, Zheng Zhao, Juan Wang, Conglong Qiu, Longhui Li, Changchun Xiong, Shanshan Zhu, Chengying Zheng

**Affiliations:** ^1^Department of Psychiatry, Affiliated Kangning Hospital of Ningbo University, Ningbo, Zhejiang, China; ^2^Department of Psychiatry, Ningbo Kangning Hospital, Ningbo, Zhejiang, China; ^3^Medical Department, Research Institute of Medical and Biological Engineering, Ningbo University, Ningbo, Zhejiang, China

**Keywords:** Alzheimer's disease, mild cognitive impairment, late-life depression, enzyme-linked immunosorbent assay, surface-enhanced Raman spectroscopy

## Abstract

**Background:**

Objective tests that can be used to identify neurodegenerative diseases and neuropsychiatric disorders are urgently needed. The primary objective of this study is to evaluate the diagnostic accuracy of surface-enhanced Raman spectroscopy (SERS), a novel blood-based detection method, in differentiating neurodegenerative diseases and neuropsychiatric disorders. Additionally, we aim to assess the synergistic diagnostic performance of combining SERS with enzyme-linked immunosorbent assay (ELISA) technology for Alzheimer's disease (AD), mild cognitive impairment (MCI), and late-life depression (LLD).

**Methods:**

In total, 23 patients with AD, 24 with MCI, 20 with LLD, and 20 cognitively normal (control) individuals were enrolled. ELISA and SERS were used to test various biomarkers in the AD, MCI, LLD, and control groups.

**Results:**

Amyloid-β, tau, brain-derived neurotrophic factor, proinflammatory cytokine IL-1β, and growth differentiation factor-15 levels as measured using ELISA significantly differed among the four groups (*P* < 0.05). SERS peaks at 592 (*P* = 0.038), 725 (*P* = 0.001), 1,003 (*P* = 0.010), 1,331 (*P* = 0.000), and 165 cm^−1^ (*P* = 0.000) likewise significantly differed among the four groups. The area under the curve was significantly higher after combining multiple blood indicators than that with single-blood indicators.

**Conclusions:**

Combining SERS and ELISA can significantly increase diagnostic accuracy for AD, MCI, and LLD. The findings are expected to provide potential therapeutic targets for precise intervention in these diseases, thereby contributing to improved clinical stratification and personalized treatment strategies.

**Clinical trial registry number:**

ChiCTR2300076307 (30/09/2023).

## Background

Age-related neurodegenerative diseases and neuropsychiatric disorders significantly increase with increased aging population (1). Cognitive decline is a common clinical manifestation of neurodegenerative diseases, such as Alzheimer's disease (AD) and neuropsychiatric disorder of late-life depression (LLD) (2). Mild cognitive impairment (MCI) is a common preclinical manifestation of neurodegenerative diseases and neuropsychiatric disorders in older adults (3, 4). In addition to negative emotions and sleep disorders, patients with LLD usually show a pseudo-cognitive and transient decline in thinking and memory, causing misdiagnosis (5, 6).

Several cognitive and psychological tests are used to evaluate cognitive levels (7, 8). Cerebrospinal fluid biomarker analysis may aid in differentiating between AD and LLD (9). Similarly, blood biomarkers are valuable objective indicators (10, 11). The transitional phase between MCI and AD is an ambiguous diagnostic period, where it is unclear whether the occurrence of MCI is owing to depression or dementia. Cognitive decline can be used to predict incipient dementia, indicating the need for different clinical treatment options. Neuropsychiatric symptoms may accompany these pre-dementia syndromes and help in identifying incipient dementia. Depression may be associated with an increased risk of dementia, particularly in older adults (12). LLD is controllable and curable if promptly diagnosed and appropriately treated. However, the onset of LLD is usually overlooked or covered up by cognitive disorders. Identifying neurodegenerative diseases and neuropsychiatric disorders using objective indicators is essential.

Enzyme-linked immunosorbent assay (ELISA) is a popular method for testing biomarkers in the blood of patients with AD. It can be used to detect serum amyloid-β (Aβ) and tau proteins (13–15). In addition, surface-enhanced Raman spectroscopy (SERS) has been recently used to investigate the diagnosis of diseases such as cancer and neurodegenerative diseases (16–18). SERS is a rapid, low-cost, non-invasive, and label-free technique that has found widespread application in *in situ* and *ex situ* biomedical diagnostics, including for neurological disorders (17). Because most previous studies used label-free SERS without specific target labeling, designing an optimal data pre-processing and modeling procedure is paramount for analyzing and interpreting untargeted spectral data. Machine learning (ML) models, such as principal component analysis, partial least squares, support vector machine, and *k*-nearest neighbors, are the prevailing methods for Raman feature extraction and data modeling (19). However, in the biomedical field, these traditional and cumbersome methods may hinder feature extraction and the identification of intricate patterns in high-dimensional Raman data, since identification or classification problems are complex tasks in practical applications. In recent years, deep learning (DL), an end-to-end learning method, has shown excellent ability in data pre-processing, feature extraction, and modeling (20). DL-based chemometrics have been applied to Raman spectral data, including cancer detection and genotype screening (21, 22).

A previous study conducted an in depth investigation of Raman spectroscopy of blood serum for AD, MCI, and other types of dementia (23). Raman spectroscopic-, SERS-, and blood or cerebrospinal fluid-based tests may aid clinical assessments, facilitating the accurate and effective differential diagnosis of AD (24, 25). In this study, we expanded the application of SERS to neuropsychiatric as well as neurodegenerative disorders and compared its diagnostic accuracy with that of ELISA. We performed two blood tests, ELISA and SERS, to investigate prompt and accurate diagnosis. We studied a combination of the two methods to collect blood from patients only once, reducing the number of invasive tests while improving diagnostic accuracy.

## Methods

### Participants

We recruited 23 patients with AD, 24 with MCI, 20 with LLD, and 20 cognitively normal participants (healthy control, HC) for this study. Patients were diagnosed using the Diagnostic and Statistical Manual of Mental Disorders, fifth edition criteria (26). All patients met the following inclusion and exclusion criteria: (1) diagnosis by at least two research psychiatrists; (2) provision of informed consent; (3) disease course >3 months; (4) presence of no other severe mental illnesses, including schizophrenia and delirium; and (5) no severe physical diseases. The Minimum sample size was calculated by setting the significance level (α) to 0.05 and the statistical power (1–β) to 70%.

### Neuropsychiatric evaluation

The Mini-Mental State Examination (MMSE) comprises cognitive questions in orientation, immediate recall, attention, short-term memory, language, and visuospatial ability (27). The MMSE has a maximum score of 30 points, with higher scores indicating better cognitive performance. A score of <22 represents patients with AD, >22 and <27 represents patients with MCI, and >27 represents cognitively normal participants. The Hamilton depression scale (HAMD) was adopted to evaluate depression in patients with LLD (28).

### Blood ELISA

Approximately 5 ml of whole blood was collected from each patient before breakfast in a procoagulant tube. The blood samples were centrifuged at 3,000 rpm for 10 min using a BY-600A type medical centrifuge (Beijing Baiyang Medical Devices Co., China). All blood samples were processed within 30 min of collection and immediately frozen at −80°C. Serum Aβ (total Aβ, Aβ_40_, and Aβ_42_), tau (total and phosphorylated), brain-derived neurotrophic factor (BDNF), proinflammatory cytokine IL-1β, and growth differentiation factor-15 (GDF-15) levels were estimated using ELISA kits (Shanghai Yuanye Bio-Technology Co., China). All procedures were performed according to the manufacturer's instructions. The absorbance was measured at 450 nm using a Sunrise-basic enzyme labeling instrument (Tecan Co., Switzerland) with a reference wavelength of 690 nm. These measurements were transformed into concentrations by comparing the optical densities of the samples with standard curve values.

### Blood SERS

For SERS measurements in this study, a substrate of the core-shell Au@Ag nanoparticles aggregates (Au@AgNA) colloidal solution was prepared through Ag deposition on the surface of Au using the seed-growth method described in a previous study (29). The thawed serum was mixed with Au@AgNAs in a 1:1 ratio and incubated for 2 h at room temperature. Furthermore, 2 μl of the mixture was dropped onto an aluminum substrate for SERS measurements. The sample was dried before SERS measurements. The SERS spectra were obtained using a RenishawinViaQontor confocal Raman spectrometer (Renishaw, UK) coupled to a Leica microscope with a 50 × objective (NA = 0.50) backscattering geometry. SERS signal was excited by a 785-nm laser and measured in a wave number range of 400−1,800 cm^−1^ with a spectral resolution of 1 cm^−1^. To reduce operational variations and repeatability errors as well as the coffee-ring effect during drying, the mean of five measurements at different positions served as the final SERS spectrum for each participant (30).

### Statistical analysis

For statistical analysis, data are presented as mean ± standard deviation. Demographic and clinical variables were compared and analyzed between the different groups using analysis of variance (ANOVA) or *t*-test for continuous variables and chi-squared test for categorical variables. SERS spectra are baseline subtracted and normalized using Savitzky–Golay algorithm as described in our previous literature (30, 31). Pearson's correlation coefficient was used to determine the correlation between cognitive levels and blood parameters. Statistical significance was set at *P* < 0.05. Receiver operating characteristic (ROC) curves and the area under the ROC curve (AUC) were used as criteria to evaluate the performance. The AUC was calculated as a comprehensive measure of discrimination accuracy, with 95% confidence intervals determined through bootstrap methods. Optimal cutoff values were identified by maximizing Youden's index (*J* = sensitivity + specificity−1).

## Results

### Clinical assessment

**Figure 1:**
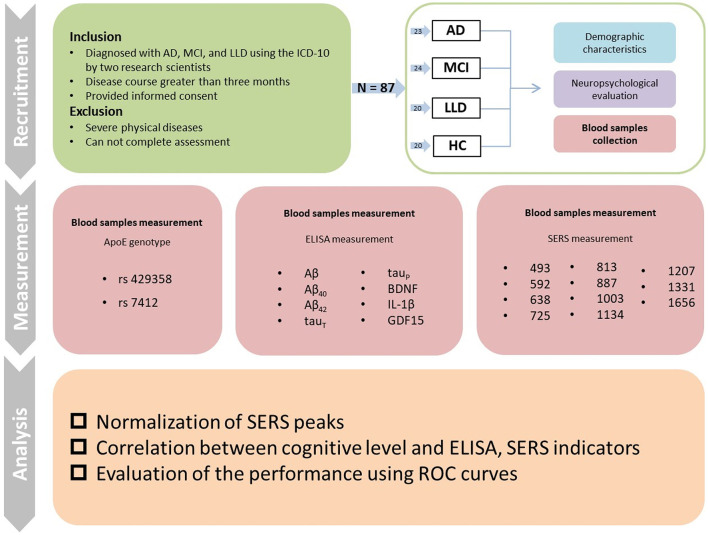
Overview of the study and CONSORT diagram of the primary phases of the clinical trial.

An overview of this study is shown in Figure 1. Inclusion and exclusion criteria were used to recruit participants with AD, MCI, LLD, and HC. All participants were asked to complete the demographic characteristics, neuropsychological evaluations, and blood sample collection. Subsequently, blood samples were analyzed using ELISA and SERS along with the APOE genotype (rs429358 and rs7412).

Table 1 presents the demographic characteristics of the participants. There were 67 patients, including 23 with AD (seven males and 16 females, with a mean age of 80.09 years), 24 with MCI (11 males and 13 females, with a mean age of 79.04 years), 20 with LLD (three males and 17 females, with a mean age of 74.10 years), and 20 HC (four males and 16 females, with a mean age of 76.00 years). Similarly, the education years, MMSE scores, HAMD scores and APOE SNPs are listed in Table 1. Allele C (TC + CC) was a risk factor for cognitive decline in the cognitive decline (MCI and AD groups) and cognitively normal (LLD and HC groups) groups at the SNP locus rs429358 (*P* < 0.05). However, there were no significant differences between the distribution of alleles C and T at locus s7412 (*P* = 0.846).

**Table 1:** Demographic characteristic of the participants.

**Items**	**AD (*n* = 23)**	**MCI (*n* = 24)**	**LLD (*n* = 20)**	**HC (*n* = 20)**	***F*/χ^2^**	***P*-value**
Age	80.09 ± 9.39	79.04 ± 7.78	74.10 ± 6.34	76.00 ± 8.00	2.536	0.062
Gender (male/female)	7/16	11/13	3/17	4/16	6.046	0.109
Education years	5.74 ± 2.91	6.00 ± 2.78	6.55 ± 2.58	7.40 ± 3.28	1.363	0.260
MMSE scores	13.17 ± 6.57	26.00 ± 2.64	28.00 ± 1.75	29.55 ± 0.69	87.653	0.000
HAMD scores	0.47 ± 0.85	0.67 ± 0.87	6.70 ± 2.05	0.65 ± 1.13	115.261	0.000
**APOE SNPs**						
**rs429358**						
TT, *n* (%)	15	14	16	18	5.031	0.027
TC, *n* (%)	3	5	4	2		
CC, *n* (%)	5	5	0	0		
**rs7412**						
TT, *n* (%)	1	0	0	0	0.038	0.846
TC, *n* (%)	2	3	3	2		
CC, *n* (%)	20	21	17	18		

Means ± SDs. AD, Alzheimer's disease; MCI, mild cognitive impairment; LLD, late life depression; HC, healthy controls; MMSE, Mini-Mental State Examination; HAMD, Hamilton depression scale; APOE, apolipoprotein E; SNP, single nucleotide polymorphism.

### Two measurements of blood biomarkers

Table 2 presents the blood levels of Aβ (total Aβ, Aβ_40_, and Aβ_42_), tau (total and phosphorylated), BDNF, proinflammatory cytokine IL-1β, and GDF-15. The levels of these blood biomarkers, measured using ELISA, significantly differed among the four groups (*P* < 0.05).

**Table 2:** Biomarkers in serum measured by ELISA.

**Items**	**AD (*n* = 23)**	**MCI (*n* = 24)**	**LLD (*n* = 20)**	**HC (*n* = 20)**	***F***	***P*-value**
Aβ (ng/ml)	452.91 ± 52.59	372.72 ± 57.41	392.21 ± 63.48	274.97 ± 83.99	36.64	0.000
Aβ_40_ (pg/ml)	407.91 ± 76.60	322.50 ± 60.45	323.89 ± 56.62	204.25 ± 52.16	37.87	0.000
Aβ_42_ (pg/ml)	691.08 ± 77.52	616.66 ± 81.36	614.92 ± 89.21	386.16 ± 76.88	55.09	0.000
tau_T_ (pg/ml)	237.24 ± 27.99	192.64 ± 28.97	186.37 ± 30.05	125.03 ± 27.80	54.86	0.000
tau_P_ (pg/ml)	309.51 ± 43.12	271.41 ± 48.32	278.20 ± 48.71	146.92 ± 38.93	52.41	0.000
BDNF (ng/ml)	7.19 ± 1.76	8.88 ± 1.74	8.63 ± 1.77	14.55 ± 1.75	70.91	0.000
IL-1β (pg/ml)	85.46 ± 9.06	70.92 ± 9.86	75.76 ± 9.97	53.94 ± 9.37	40.04	0.000
GDF15 (pg/ml)	1,436.00 ± 210.74	1,131.06 ± 187.91	1,257.85 ± 199.37	728.97 ± 196.60	47.83	0.000

Means ± SDs. AD, Alzheimer's disease; MCI, mild cognitive impairment; LLD, late life depression; HC, healthy controls; Aβ, β-amyloid; BDNF, brain derived neurotrophic factor; GDF, growth differentiation factor.

Table 3 lists the peak positions measured using SERS for the four groups. After performing ANOVA on the intensities of the primary SERS peaks (each peak represents specific substance components), five SERS peaks at 592 (*P* = 0.038), 725 (*P* = 0.001), 1,003 (*P* = 0.010), 1,331 (*P* = 0.000), and 1,656 cm^−1^ (*P* = 0.000) had statistically significant differences among the four groups.

**Table 3:** Intensity of the primary SERS peaks of serum.

**Peak position (cm^−1^)**	**Intensity (arb. unit)**				***F***	***P*-value**	**Assignments**
	**AD (*****n*** = **23)**	**MCI (*****n*** = **24)**	**LLD (*****n*** = **20)**	**HC (*****n*** = **20)**			
493	0.493 ± 0.026	0.486 ± 0.053	0.494 ± 0.093	0.510 ± 0.019	0.741	0.530	Ring vibration, cellulose, guanine, L-arginine
592	0.307 ± 0.018	0.293 ± 0.036	0.276 ± 0.054	0.295 ± 0.015	2.946	0.038	Ascorbic acid, amide-VI
638	0.995 ± 0.012	0.975 ± 0.102	0.956 ± 0.169	0.999 ± 0.001	0.847	0.472	C-S stretching vibration, L-tyrosine, lactose
725	0.558 ± 0.222	0.427 ± 0.198	0.566 ± 0.232	0.337 ± 0.133	6.305	0.001	C-H bending vibration, adenine, coenzyme A
813	0.276 ± 0.016	0.281 ± 0.032	0.284 ± 0.053	0.296 ± 0.014	1.511	0.218	C-C-O stretching vibration, L-serine, glutathione
887	0.287 ± 0.029	0.303 ± 0.029	0.306 ± 0.054	0.288 ± 0.017	1.744	0.164	C-O-H bending vibration, glutathione, D-(+)-galactosamine
1,003	0.253 ± 0.035	0.232 ± 0.031	0.221 ± 0.055	0.216 ± 0.028	4.000	0.010	C-C symmetric stretch, phenylalanine
1,134	0.596 ± 0.055	0.589 ± 0.063	0.596 ± 0.116	0.573 ± 0.028	0.481	0.697	C-N stretching vibration, D-mannose
1,207	0.328 ± 0.032	0.326 ± 0.031	0.342 ± 0.065	0.320 ± 0.023	0.994	0.400	Ring vibration, L-tryptophan, phenylalanine
1,331	0.341 ± 0.060	0.298 ± 0.051	0.344 ± 0.055	0.233 ± 0.039	20.059	0.000	C-H stretching vibration, nucleic acid bases, D-mannose
1,656	0.722 ± 0.126	0.657 ± 0.119	0.752 ± 0.168	0.515 ± 0.083	13.916	0.000	C=O stretching vibration, amide-I, α-helix

Means ± SDs. AD, Alzheimer's disease; MCI, mild cognitive impairment; LLD, late life depression; HC, healthy controls.

**Figure 2:**
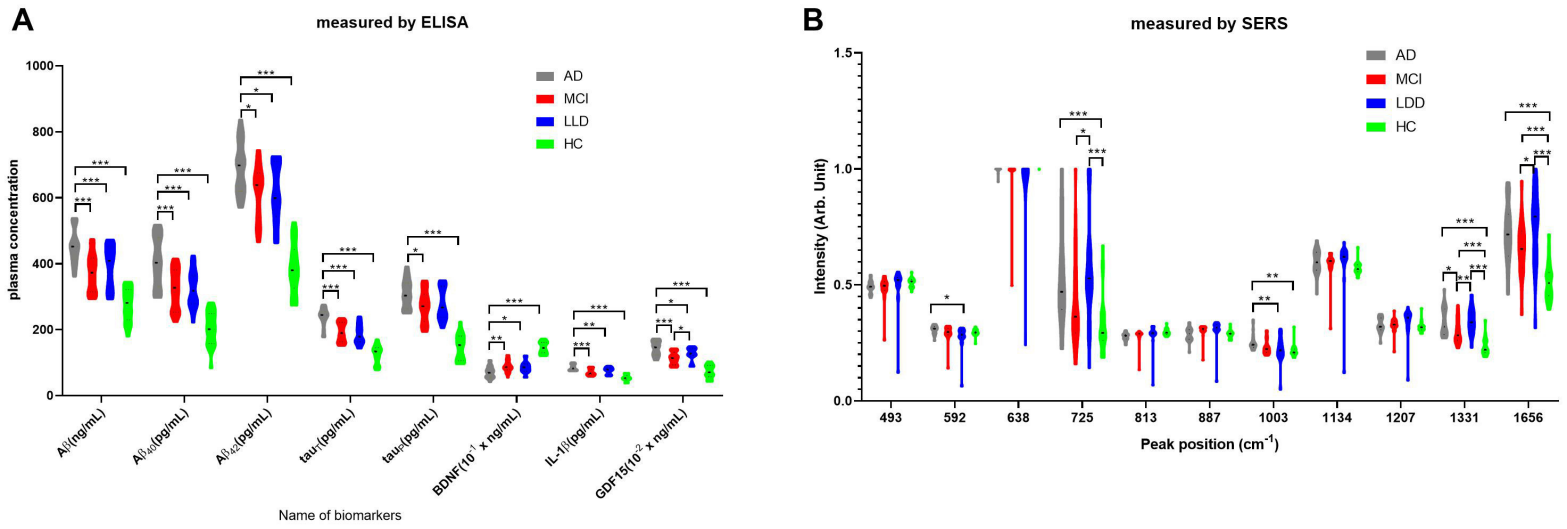
Two measurements of blood biomarkers. **(A)** Enzyme-linked immunosorbent assay (ELISA) data of total Aβ, Aβ_40_, Aβ_42_, total tau, phosphorylated tau, brain-derived neurotrophic factor (BDNF), proinflammatory cytokine IL-1β, and growth differentiation factor-15 (GDF-15). **(B)** SERS data of peaks position in 493, 592, 638, 725, 813, 887, 1,003, 1,134, 1,207, 1,331, and 1,656 cm^−1^. The gray bar represents the AD group. The red bar represents the MCI group. The blue bar represents the LLD group. The green bar represents the HC group.*P < 0.05; **P < 0.01; ***P < 0.001.

As shown in Figure 2A, using the ELISA measurement, the serum biomarkers were significantly higher in the AD group than in the other three groups. Compared with the MCI group, the peak positions of SERS (in terms of intensity) at 725, 1,331, and 1,656 cm^−1^ had statistically significant differences in the LLD group (Figure 2B).

**Figure 3:**
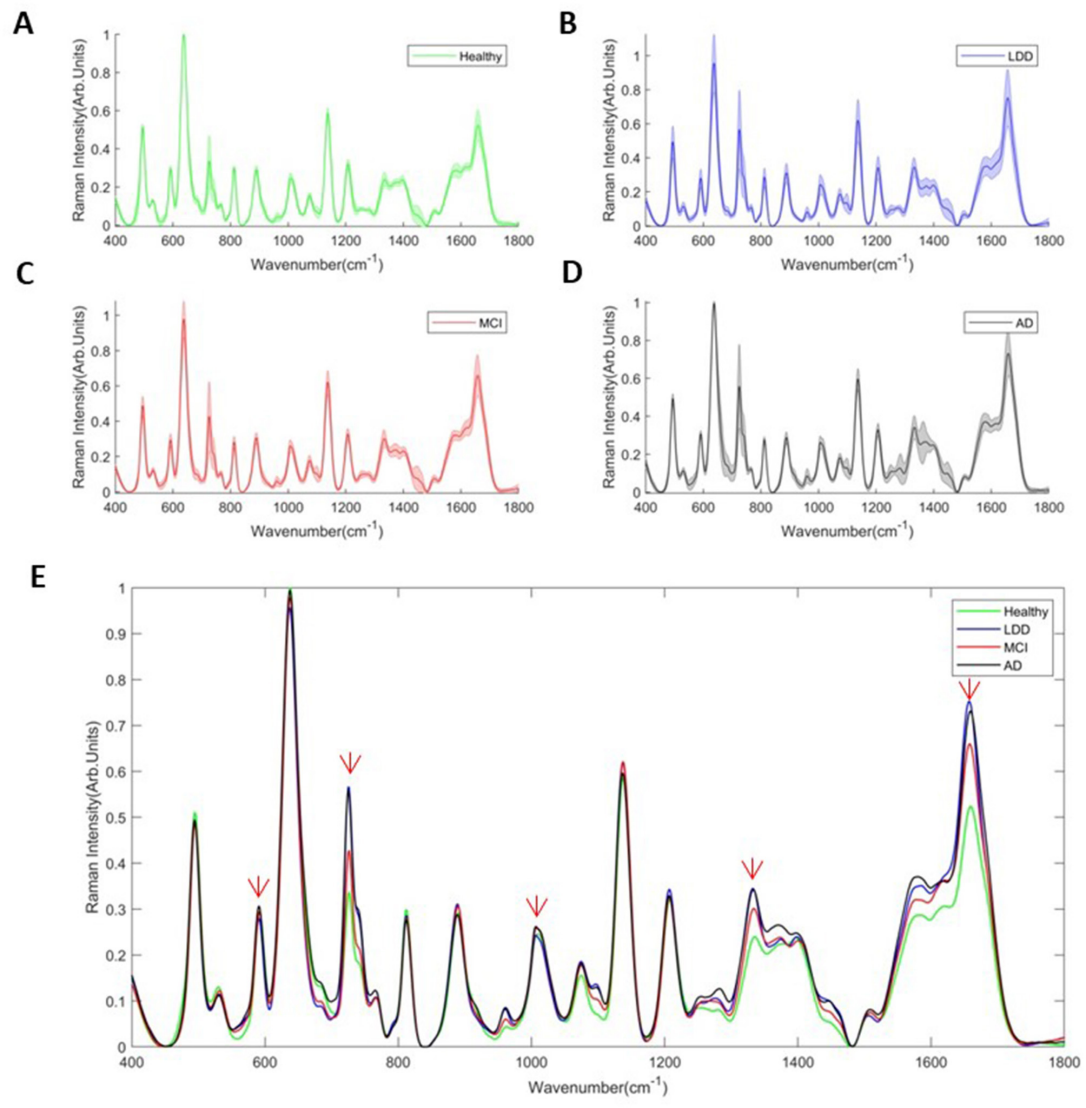
Intensity of the average serum SERS spectra of the AD **(A)**, MCI **(B)**, LLD **(C)**, and HC groups **(D)** and the comparison of average serum SERS spectra between four groups **(E)**. The gray line represents the AD group. The red line represents the MCI group. The blue line represents the LLD group. The green line represents the HC group.

Figures 3A–D shows the average serum SERS spectra of the HC (Figure 3A), LLD (Figure 3B), MCI (Figure 3C), and AD (Figure 3D) groups and the comparison of the average serum SERS spectra between the four groups (Figure 3E). Similarly, Figure 3E shows SERS intensities with statistically significant differences between the four groups at peaks 592, 725, 1,003, 1,331, and 1,656 cm^−1^.

### Correlations between two types of blood biomarkers

**Figure 4:**
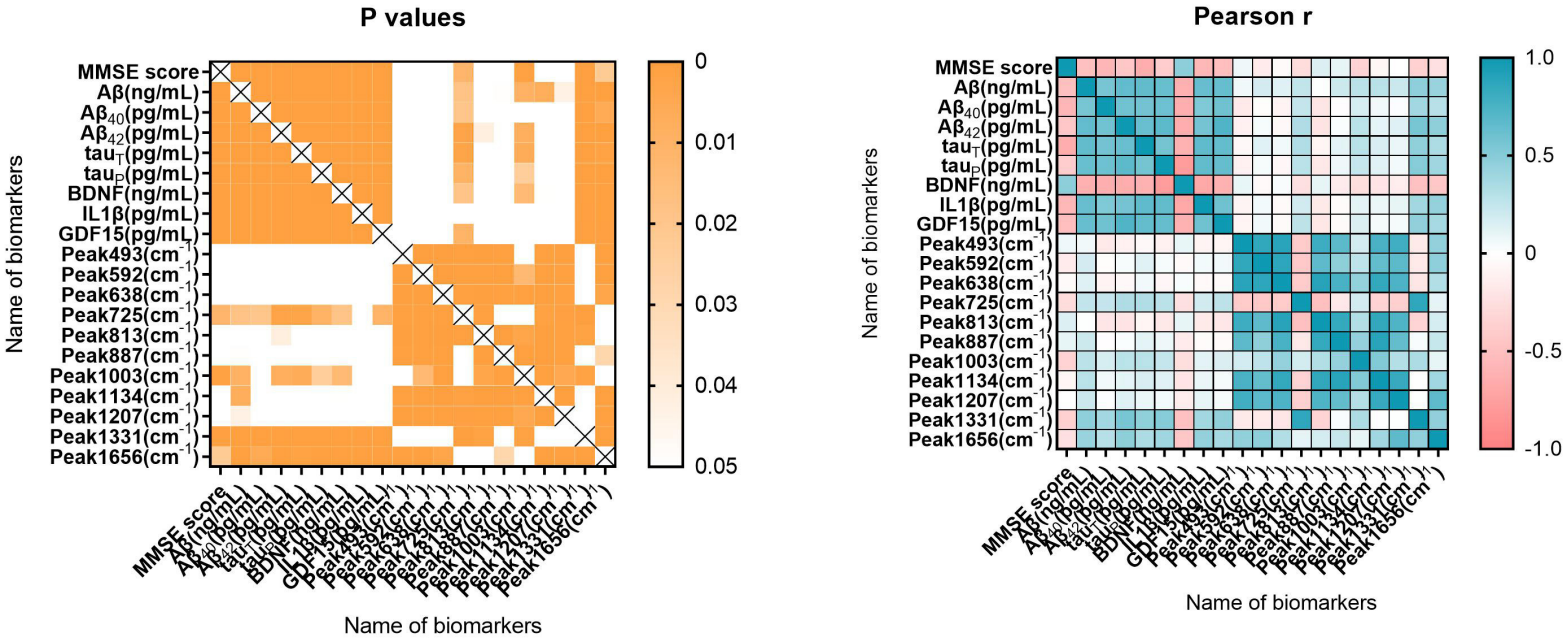
Pearson correlation of cognitive and blood biomarkers. MMSE represents the cognitive level of participants. The blood parameters included ELISA measurements of (total Aβ, Aβ40, and Aβ42), tau (including total and phosphorylated tau), brain-derived neurotrophic factor (BDNF), proinflammatory cytokine IL-1β, growth differentiation factor-15 (GDF-15) and SERS measurements of peaks position in 493, 592, 638, 725, 813, 887, 1,003, 1,134, 1,207, 1,331, and 1,656 cm^−1^. The scale bar represents Pearson's correlation coefficient *r* and *P*-values.

Figure 4 shows the Pearson correlation between cognitive and blood biomarkers. MMSE scores were significantly correlated with Aβ (*P* < 0.001, *r* = −0.498), Aβ_40_ (*P* < 0.001, *r* = −0.579), Aβ_42_ (*P* < 0.001, *r* = −0.445), total tau (*P* < 0.001, *r* = −0.638), phosphorylated tau (*P* < 0.001, *r* = −0.396), BDNF (*P* < 0.001, *r* = 0.474), proinflammatory cytokine IL-1β (*P* < 0.001, *r* = −0.560), and GDF-15 (*P* < 0.001, *r* = −0.509). Regarding SERS parameters, MMSE scores were significantly correlated with 725 (*P* = 0.011, *r* = −0.271), 1,003 (*P* = 0.001, *r* = −0.348), 1,331 (*P* < 0.001, *r* = −0.366), and 1,656 cm^−1^ (*P* = 0.022, *r* = −0.245).

As presented in Table 4, all individuals with APOE rs429358 T/T genotype had lower Aβ_40_ and Aβ_42_ and higher BDNF levels than those with APOE rs429358 T/C + C/C genotype (*t* = −2.060, *P* = 0.042; *t* = −2.290, *P* = 0.026; *t* = 2.573, *P* = 0.012, respectively). Compared with APOE rs429358 T/C + C/C genotype carriers, individuals with the APOE rs429358 T/T genotype showed no significant differences in SERS data. In contrast to individuals with the APOE rs7412 C/C genotype, those with the APOE rs7412 T/C + T/T genotype showed no significant differences in ELISA and SERS data (Table 5).

**Table 4:** Blood parameters of ELISA and SERS to APOE rs429358 in all participants.

**Biomarkers**	**rs429358**		***t***	***P*-value**
	**T/T (*****n*** = **63)**	**T/C** + **C/C (*****n*** = **24)**		
**ELISA**				
Aβ (ng/ml)	375.91 ± 87.35	375.99 ± 76.24	−0.005	0.996
Aβ_40_ (pg/ml)	305.54 ± 91.00	351.50 ± 98.12	−2.060	0.042
Aβ_42_ (pg/ml)	564.99 ± 145.01	630.08 ± 106.67	−2.290	0.026
tau_T_ (pg/ml)	183.12 ± 49.45	198.81 ± 45.73	−1.350	0.181
tau_P_ (pg/ml)	249.67 ± 81.29	266.91 ± 56.24	−1.121	0.267
BDNF (ng/ml)	10.12 ± 3.51	8.53 ± 2.11	2.573	0.012
IL-1β (pg/ml)	70.54 ± 15.46	75.72 ± 12.00	−1.477	0.143
GDF15 (pg/ml)	1,111.98 ± 323.91	1,243.97 ± 304.96	−1.726	0.088
**SERS**				
Peak 493 (cm^−1^)	0.50 ± 0.04	0.49 ± 0.08	0.812	0.419
Peak 592 (cm^−1^)	0.29 ± 0.03	0.29 ± 0.05	0.086	0.931
Peak 638 (cm^−1^)	0.99 ± 0.06	0.97 ± 0.15	0.948	0.346
Peak 725 (cm^−1^)	0.46 ± 0.23	0.50 ± 0.19	−0.809	0.421
Peak 813 (cm^−1^)	0.29 ± 0.03	0.27 ± 0.05	1.655	0.102
Peak 887 (cm^−1^)	0.30 ± 0.03	0.29 ± 0.05	0.837	0.405
Peak 1,003 (cm^−1^)	0.23 ± 0.03	0.24 ± 0.06	−0.929	0.355
Peak 1,134 (cm^−1^)	0.59 ± 0.05	0.58 ± 0.11	0.643	0.522
Peak 1,207 (cm^−1^)	0.33 ± 0.03	0.33 ± 0.06	0.303	0.762
Peak 1,331 (cm^−1^)	0.30 ± 0.07	0.32 ± 0.06	−1.328	0.188
Peak 1,656 (cm^−1^)	0.65 ± 0.15	0.70 ± 0.16	−1.520	0.132

Means ± SDs. Aβ, β-amyloid; BDNF, brain derived neurotrophic factor; GDF, growth differentiation factor.

**Table 5:** Blood parameters of ELISA and SERS to APOE rs7412 in all participants.

**Biomarkers**	**rs7412**		***t***	***P*-value**
	**T/T** + **T/C (*****n*** = **11)**	**C/C (*****n*** = **76)**		
**ELISA**				
Aβ (ng/ml)	412.04 ± 85.36	370.70 ± 83.06	1.538	0.128
Aβ_40_ (pg/ml)	332.83 ± 89.61	316.10 ± 95.82	0.545	0.587
Aβ_42_ (pg/ml)	605.59 ± 114.95	579.67 ± 141.44	0.580	0.564
tau_T_ (pg/ml)	173.31 ± 42.13	189.49 ± 49.49	−1.030	0.306
tau_P_ (pg/ml)	289.37 ± 75.55	249.37 ± 74.40	1.664	0.100
BDNF (ng/ml)	8.73 ± 3.58	9.82 ± 3.20	−1.038	0.302
IL-1β (pg/ml)	74.06 ± 13.38	71.69 ± 14.94	0.502	0.617
GDF15 (pg/ml)	1,129.09 ± 278.22	1,151.18 ± 330.00	−0.211	0.833
**SERS**				
Peak 493 (cm^−1^)	0.50 ± 0.02	0.49 ± 0.06	0.528	0.599
Peak 592 (cm^−1^)	0.30 ± 0.01	0.29 ± 0.04	0.949	0.345
Peak 638 (cm^−1^)	0.99 ± 0.01	0.98 ± 0.10	0.533	0.595
Peak 725 (cm^−1^)	0.43 ± 0.21	0.48 ± 0.22	−0.775	0.440
Peak 813 (cm^−1^)	0.29 ± 0.01	0.28 ± 0.03	0.552	0.582
Peak 887 (cm^−1^)	0.29 ± 0.02	0.30 ± 0.04	0.237	0.814
Peak 1,003 (cm^−1^)	0.22 ± 0.02	0.23 ± 0.04	−0.984	0.328
Peak 1,134 (cm^−1^)	0.59 ± 0.03	0.58 ± 0.08	0.516	0.607
Peak 1,207 (cm^−1^)	0.34 ± 0.03	0.32 ± 0.04	1.118	0.267
Peak 1,331 (cm^−1^)	0.29 ± 0.05	0.31 ± 0.07	−0.532	0.596
Peak 1,656 (cm^−1^)	0.73 ± 0.17	0.65 ± 0.15	1.607	0.112

Means ± SDs. Aβ, β-amyloid; BDNF, brain derived neurotrophic factor; GDF, growth differentiation factor.

Table 6 shows the relation of the ELISA and SERS measurements of blood parameters with APOE rs429358 in MCI participants. The data on APOE rs429358 T/T and APOE rs429358 T/C + C/C genotype carriers did not significantly differ from the ELISA and SERS results. Table 7 shows the relation of the blood parameters measured using ELISA and SERS with APOE rs7412 in MCI participants. Compared with individuals with the APOE rs7412 C/C genotype, those with the APOE rs7412 T/C + T/T genotype showed a significantly higher level of tau_p_ value (*t* = 1.788, *P* = 0.016) on ELISA and lower value of Peak 725 on SERS (*t* = −1.119, *P* = 0.038).

**Table 6:** Blood parameters of ELISA and SERS to APOE rs429358 in MCI participants.

**Biomarkers**	**rs429358**		***t***	***P*-value**
	**T/T (*****n*** = **14)**	**T/C** + **C/C (*****n*** = **10)**		
**ELISA**				
Aβ (ng/ml)	360.60 ± 56.07	381.38 ± 58.83	−0.870	0.394
Aβ_40_ (pg/ml)	316.85 ± 57.98	330.40 ± 66.04	−0.533	0.599
Aβ_42_ (pg/ml)	596.94 ± 80.48	630.74 ± 81.94	−1.004	0.326
tau_T_ (pg/ml)	188.27 ± 28.46	195.76 ± 29.99	−0.615	0.542
tau_P_ (pg/ml)	269.98 ± 34.95	272.43 ± 57.30	−0.120	0.898
BDNF (ng/ml)	9.42 ± 1.73	8.12 ± 1.52	1.902	0.065
IL-1β (pg/ml)	70.62 ± 9.25	71.33 ± 11.16	−0.170	0.866
GDF15 (pg/ml)	1,128.78 ± 233.59	1,132.69 ± 157.12	−0.046	0.961
**SERS**				
Peak 493 (cm^−1^)	0.50 ± 0.03	0.48 ± 0.07	1.126	0.218
Peak 592 (cm^−1^)	0.30 ± 0.02	0.28 ± 0.04	1.399	0.176
Peak 638 (cm^−1^)	0.99 ± 0.01	0.96 ± 0.13	0.807	0.428
Peak 725 (cm^−1^)	0.43 ± 0.14	0.43 ± 0.23	−0.023	0.982
Peak 813 (cm^−1^)	0.29 ± 0.01	0.28 ± 0.04	0.714	0.482
Peak 887 (cm^−1^)	0.31 ± 0.01	0.30 ± 0.04	0.926	0.365
Peak 1,003 (cm^−1^)	0.22 ± 0.03	0.24 ± 0.03	−1.598	0.124
Peak 1,134 (cm^−1^)	0.60 ± 0.02	0.58 ± 0.08	0.633	0.533
Peak 1,207 (cm^−1^)	0.32 ± 0.03	0.34 ± 0.03	−1.332	0.177
Peak 1,331 (cm^−1^)	0.30 ± 0.06	0.30 ± 0.05	−0.174	0.864
Peak 1,656 (cm^−1^)	0.63 ± 0.12	0.69 ± 0.11	−1.202	0.242

Means ± SDs. Aβ, β-amyloid; BDNF, brain derived neurotrophic factor; GDF, growth differentiation factor.

**Table 7:** Blood parameters of ELISA and SERS to APOE rs7412 in MCI participants.

**Biomarkers**	**rs7412**		***t***	***P*-value**
	**T/T** + **T/C (*****n*** = **3)**	**C/C (*****n*** = **21)**		
**ELISA**				
Aβ (ng/ml)	431.85 ± 41.95	364.27 ± 54.90	2.033	0.045
Aβ_40_ (pg/ml)	346.69 ± 33.34	319.04 ± 63.17	0.734	0.302
Aβ_42_ (pg/ml)	617.43 ± 81.65	611.23 ± 97.02	0.121	0.905
tau_T_ (pg/ml)	193.49 ± 29.57	186.68 ± 29.10	0.374	0.712
tau_P_ (pg/ml)	315.98 ± 20.01	265.04 ± 48.00	1.788	0.016
BDNF (ng/ml)	8.43 ± 1.24	8.94 ± 1.81	−0.471	0.642
IL-1β (pg/ml)	74.12 ± 14.00	70.46 ± 9.51	0.593	0.559
GDF15 (pg/ml)	1,050.05 ± 83.91	1,142.63 ± 196.91	−0.792	0.203
**SERS**				
Peak 493 (cm^−1^)	0.49 ± 0.01	0.48 ± 0.06	0.453	0.655
Peak 592 (cm^−1^)	0.30 ± 0.02	0.29 ± 0.04	0.217	0.830
Peak 638 (cm^−1^)	0.99 ± 0.01	0.97 ± 0.11	0.360	0.722
Peak 725 (cm^−1^)	0.31 ± 0.06	0.44 ± 0.21	−1.119	0.038
Peak 813 (cm^−1^)	0.29 ± 0.01	0.28 ± 0.03	0.554	0.159
Peak 887 (cm^−1^)	0.31 ± 0.01	0.30 ± 0.03	0.168	0.710
Peak 1,003 (cm^−1^)	0.22 ± 0.01	0.23 ± 0.03	−0.896	0.103
Peak 1,134 (cm^−1^)	0.61 ± 0.03	0.59 ± 0.07	0.543	0.387
Peak 1,207 (cm^−1^)	0.34 ± 0.02	0.33 ± 0.03	0.574	0.572
Peak 1,331 (cm^−1^)	0.27 ± 0.01	0.30 ± 0.05	−0.932	0.050
Peak 1,656 (cm^−1^)	0.70 ± 0.10	0.65 ± 0.12	0.596	0.558

Means ± SDs. Aβ, β-amyloid; BDNF, brain derived neurotrophic factor; GDF, growth differentiation factor.

Table 8 compares the blood parameters measured using ELISA and SERS with respect to APOE rs429358 in LLD participants. Compared with individuals with the APOE rs429358 T/T genotype, those with the APOE rs429358 T/C + C/C genotype showed significantly higher Aβ and GDF15 levels (*t* = −2.176, *P* = 0.043; *t* = −1.808, *P* = 0.021, respectively) on ELISA and a significantly higher value of Peak 725 (*t* = −0.972, *P* = 0.025) on SERS. Table 9 shows the association of the blood parameters measured using ELISA and SERS with APOE rs7412 in LLD participants. Compared with individuals with the APOE rs7412 C/C genotype, those with the APOE rs7412 T/C + T/T genotype showed significantly higher levels of tau_T_ (*t* = 1.644, *P* = 0.002) on ELISA and higher values of Peak 592 and 1,656 (*t* = 1.026, *P* = 0.030; *t* = 2.499, *P* = 0.025, respectively) and lower value of Peak 1,331 (*t* = −0.737, *P* = 0.015) on SERS.

**Table 8:** Blood parameters of ELISA and SERS to APOE rs429358 in LLD participants.

**Biomarkers**	**rs429358**		***t***	***P*-value**
	**T/T (*****n*** = **16)**	**T/C** + **C/C (*****n*** = **4)**		
**ELISA**				
Aβ (ng/ml)	335.73 ± 64.81	406.33 ± 56.58	−2.176	0.043
Aβ_40_ (pg/ml)	322.15 ± 61.18	330.91 ± 38.88	−0.270	0.790
Aβ_42_ (pg/ml)	605.08 ± 91.88	654.25 ± 74.90	−0.985	0.338
tau_T_ (pg/ml)	179.22 ± 33.71	188.16 ± 29.98	−0.522	0.608
tau_P_ (pg/ml)	277.33 ± 68.75	278.42 ± 45.38	−0.039	0.969
BDNF (ng/ml)	9.40 ± 1.78	8.44 ± 1.77	0.970	0.345
IL-1β (pg/ml)	70.78 ± 10.33	77.00 ± 9.81	−1.123	0.276
GDF15 (pg/ml)	1,219.77 ± 201.83	1,410.19 ± 96.86	−1.808	0.021
**SERS**				
Peak 493 (cm^−1^)	0.51 ± 0.03	0.43 ± 0.21	1.481	0.528
Peak 592 (cm^−1^)	0.29 ± 0.02	0.23 ± 0.11	1.961	0.407
Peak 638 (cm^−1^)	0.99 ± 0.03	0.81 ± 0.40	2.082	0.052
Peak 725 (cm^−1^)	0.54 ± 0.23	0.67 ± 0.23	−0.972	0.025
Peak 813 (cm^−1^)	0.30 ± 0.02	0.23 ± 0.11	2.437	0.344
Peak 887 (cm^−1^)	0.32 ± 0.01	0.25 ± 0.11	2.444	0.333
Peak 1,003 (cm^−1^)	0.23 ± 0.04	0.19 ± 0.10	1.318	0.204
Peak 1,134 (cm^−1^)	0.62 ± 0.04	0.50 ± 0.25	2.003	0.060
Peak 1,207 (cm^−1^)	0.35 ± 0.03	0.29 ± 0.14	1.483	0.155
Peak 1,331 (cm^−1^)	0.34 ± 0.06	0.37 ± 0.02	−1.185	0.054
Peak 1,656 (cm^−1^)	0.76 ± 0.14	0.73 ± 0.28	0.319	0.753

Means ± SDs. Aβ, β-amyloid; BDNF, brain derived neurotrophic factor; GDF, growth differentiation factor.

**Table 9:** Blood parameters of ELISA and SERS to APOE rs7412 in LLD participants.

**Biomarkers**	**rs7412**		***t***	***P*-value**
	**T/T** + **T/C (*****n*** = **3)**	**C/C (*****n*** = **17)**		
**ELISA**				
Aβ (ng/ml)	406.06 ± 62.74	389.75 ± 65.19	0.401	0.693
Aβ_40_ (pg/ml)	289.43 ± 63.21	329.98 ± 55.19	−1.153	0.264
Aβ_42_ (pg/ml)	637.48 ± 75.00	610.93 ± 92.92	0.465	0.647
tau_T_ (pg/ml)	190.81 ± 30.45	161.18 ± 6.22	1.644	0.002
tau_P_ (pg/ml)	296.76 ± 52.26	274.92 ± 48.98	0.706	0.489
BDNF (ng/ml)	7.23 ± 1.68	8.88 ± 1.72	−1.535	0.142
IL-1β (pg/ml)	75.88 ± 9.99	75.02 ± 12.01	0.136	0.894
GDF15 (pg/ml)	1,274.70 ± 193.98	1,162.37 ± 245.95	0.895	0.383
**SERS**				
Peak 493 (cm^−1^)	0.52 ± 0.02	0.49 ± 0.09	0.649	0.496
Peak 592 (cm^−1^)	0.31 ± 0.01	0.27 ± 0.06	1.026	0.030
Peak 638 (cm^−1^)	0.99 ± 0.01	0.95 ± 0.18	0.452	0.288
Peak 725 (cm^−1^)	0.42 ± 0.11	0.59 ± 0.23	−1.228	0.094
Peak 813 (cm^−1^)	0.29 ± 0.01	0.28 ± 0.06	0.348	0.424
Peak 887 (cm^−1^)	0.31 ± 0.01	0.31 ± 0.06	0.031	0.941
Peak 1,003 (cm^−1^)	0.20 ± 0.02	0.23 ± 0.06	−0.629	0.214
Peak 1,134 (cm^−1^)	0.62 ± 0.02	0.59 ± 0.12	0.434	0.327
Peak 1,207 (cm^−1^)	0.38 ± 0.02	0.33 ± 0.06	1.305	0.208
Peak 1,331 (cm^−1^)	0.32 ± 0.01	0.34 ± 0.06	−0.737	0.015
Peak 1,656 (cm^−1^)	0.95 ± 0.05	0.72 ± 0.16	2.449	0.025

Means ± SDs. Aβ, β-amyloid; BDNF, brain derived neurotrophic factor; GDF, growth differentiation factor.

### Sensitivity of two measurements in the diagnosis of MCI and LLD

**Figure 5:**
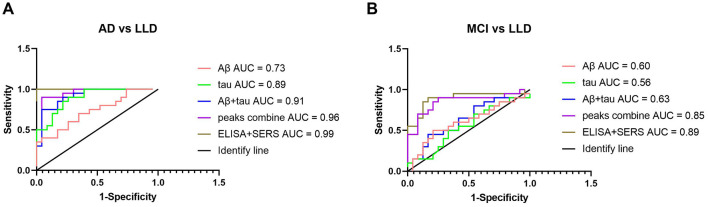
Receiver operating characteristic curves of different parameters of diagnosis in cognitive decline AD **(A)** or MCI **(B)** and no cognitive decline LLD. Combining ELISA and SERS parameters can improve the sensitivity and specificity. AD, Alzheimer's disease; MCI, mild cognitive impairment; LLD, late-life depression. The red line represents Aβ; the green line represents tau; the blue line represents the combination of Aβ and tau; the purple line represents five peaks of 592, 725, 1,003, 1,331, and 1,656 cm^−1^; the brown line represents the combination of ELISA and SERS parameters; the black line represents the identify line.

For the MCI and LLD groups, the blood parameters of the two measurements could be used to enhance the accuracy of the cognitive disorder diagnosis. In Figure 5, the ROC curve revealed that the AUC was significantly higher after combining multiple blood indicators than with a single blood indicator. The results demonstrate that SERS-based methods yielded an accuracy of 96% for the classification of AD and LLD, and an accuracy of 85% for the classification of MCI and LLD. The combination of SERS and ELISA yielded an accuracy of 99% for the classification of AD and LLD, and an accuracy of 89% for the classification of MCI and LLD.

## Discussion

This is the first study to involve using two different blood tests to investigate biomarkers for neuropsychiatric diseases. In previous studies, ELISA-SERS was used to detect the severe acute respiratory syndrome coronavirus 2 and other diseases (32, 33). In this study, we aimed to examine whether two blood tests, ELISA and SERS, could be used to differentiate between AD, MCI, and LLD better than single blood tests. The Aβ and tau biomarkers remained the elevated indicators in AD, MCI, and LLD, as previously described (34). The SERS peaks are valuable and well-established biomarkers, characterized by specific Raman fingerprints that can be used to identify AD, MCI, and LLD (30). We found that combining the two methods can improve the diagnosis and that SERS complements the ELISA.

The APOE genotype may be a genetic risk factor for neurodegenerative diseases other than Alzheimer's disease (35). Contributions of Aβ burden and APOE genotype on cognitive performance were also risk factors for cognitive decline in participants with LLD (36). The SNPs of rs429358 in APOE genotype C and rs7412 in APOE genotype T are risk factors for cognitive decline. In this study, the blood parameters Aβ_40_ and Aβ_42_ of ELISA were significantly different in participants with and without SNPs of the rs429358 APOE genotype. However, in SERS parameters, there were no statistically significant differences between participants with and without SNPs of the rs429358 and rs7412 APOE genotypes. This suggested that the SERS findings were more flexible in the absence of APOE genotypes.

In a previous study, the mean SERS spectra of different groups were the biochemical component assignments of these peaks (30). Among these peaks, the SERS intensities at 592(*P* = 0.038), 725 (*P* = 0.001), 1,003 (*P* = 0.010), 1,331 (*P* = 0.000), and 1,656 cm^−1^ (*P* = 0.000) were significantly different among the four groups, demonstrating the different biochemical components of these groups. These components included L-serine, glutathione, adenine, coenzyme A, phenylalanine, and nucleic and acid bases, among others (37–39). Changes in these components correspond to the processes in neuropsychiatric disorders, cellular metabolism, and neurological functions (40, 41).

The SERS peak at 592 cm^−1^ represented ascorbic acid and amide-VI. Ascorbic acid is a water-soluble antioxidant that catalyzes the reduction of superoxide radicals and plays a crucial role in maintaining oxidative balance (42). Many studies have indicated that ascorbic acid deficiency is associated with depression (43). Lower ascorbic acid status is also associated with greater cognitive impairment (44, 45). Increased consumption of ascorbic acid caused by oxidative stress in the brain may lead to reduced levels in serum of patients with MCI and AD (46).

The SERS peak at 725 cm^−1^ represented C-H bending vibration, adenine, and coenzyme A. Coenzyme A metabolism plays a crucial role in the normal functioning and metabolism of the nervous system (47). Inborn errors of coenzyme A metabolism are responsible for distinct forms of neurodegeneration with brain iron accumulation (47, 48). The SERS peak at 1,003 cm^−1^ represented the C-C symmetric stretch and phenylalanine. Dysregulation of phenylalanine metabolism in the hippocampus may be an important pathogenic mechanism for AD (49).

The SERS peak at 1,331 cm^−1^ represented C-H stretching vibration, nucleic acid bases, and D-mannose. The results suggested more cell-free DNA in the blood in patients with neuropsychiatric disorders. Circulating cell-free DNA is a product of cell death. The increase in circulating cell-free DNA levels might result from excessive cell death in the brain due to higher oxidative stress levels in neuropsychiatric disorders (50).

The SERS peak of 1,656 cm^−1^ represented C=O stretching vibration, amide-I, and α-helix. The results indicated increased levels of free amino acids in the blood serum of patients with neuropsychiatric disorders, which aligns with the findings of several recent studies (51). Amino acids play essential roles in controlling brain functions by acting as regulators of energy metabolism (52). Alterations in free amino acid levels in blood may be influenced by compromised energy metabolism, including nitrogen metabolism and cerebral glucose metabolism in patients with neuropsychiatric disorders (53).

The ELISA method provided information on protein levels, including Aβ (total Aβ, Aβ_40_, and Aβ_42_), tau (total and phosphorylated), BDNF, IL-1β, and GDF-15. GDF-15 is significant in the biological aging of LLD (54). Compared with patients with MCI, participants with LLD had significantly higher GDF-15 levels. Late-life depression is associated with GDF-15, a marker of age-related biological changes. As it can measure the inelastic scattering between monochromatic photons and detected molecules, SERS can provide specific information on metabolic variations, which may serve as valuable biomarkers in neurodegenerative and neuropsychiatric disorders (55, 56). Combining ELISA and SERS blood testing may be a valuable method to increase diagnostic accuracy.

This study has some limitations and strengths. The strength of this study was its elevated diagnostic accuracy using minimally invasive methods of blood testing for material composition at two different scales. A limitation of this study is that the sample size was relatively small, which lead to high intra-class standard deviations for the proposed SERS peaks. In future studies, we will endeavor to enroll larger samples or collect a larger number of SERS spectra from the same samples/donors and use advanced data analysis methods suitable for large sample processing, such as machine learning, to train classification models and increase diagnostic accuracy.

## Conclusions

Blood biomarkers tested using ELISA and SERS are associated with cognitive level. Combining ELISA and SERS is an innovative technique that can significantly increase diagnostic accuracy. Biomarkers of material composition at two scales can be used to identify age-related neurodegenerative diseases and neuropsychiatric disorders. Using the SERS + ELISA method can improve the 96% accuracy for the classification of AD and LLD to 99% for the classification of MCI and LLD. The combination of SERS and ELISA improved the accuracy of MCI and LLD classification from 85 to 89%.

## Data Availability

The raw data supporting the conclusions of this article will be made available by the authors, without undue reservation.
